# How Does Spatial Attention Influence the Probability and Fidelity of Colour Perception?

**DOI:** 10.3390/vision3020031

**Published:** 2019-06-17

**Authors:** Austin J. Hurst, Michael A. Lawrence, Raymond M. Klein

**Affiliations:** 1 Department of Psychology & Neuroscience, Dalhousie University, Halifax, NS B3H 4R2, Canada; 2 Department of Psychology, University of Waterloo, Waterloo, ON N2L 3G1, Canada

**Keywords:** visual perception, colour wheel, spatial attention, exogenous, endogenous

## Abstract

Existing research has found that spatial attention alters how various stimulus properties are perceived (e.g., luminance, saturation), but few have explored whether it improves the accuracy of perception. To address this question, we performed two experiments using modified Posner cueing tasks, wherein participants made speeded detection responses to peripheral colour targets and then indicated their perceived colours on a colour wheel. In E1, cues were central and endogenous (i.e., prompted voluntary attention) and the interval between cues and targets (stimulus onset asynchrony, or SOA) was always 800 ms. In E2, cues were peripheral and exogenous (i.e., captured attention involuntarily) and the SOA varied between short (100 ms) and long (800 ms). A Bayesian mixed-model analysis was used to isolate the effects of attention on the probability and the fidelity of colour encoding. Both endogenous and short-SOA exogenous spatial cueing improved the probability of encoding the colour of targets. Improved fidelity of encoding was observed in the endogenous but not in the exogenous cueing paradigm. With exogenous cues, inhibition of return (IOR) was observed in both RT and probability at the long SOA. Overall, our findings reinforce the utility of continuous response variables in the research of attention.

## 1. Introduction

Attention is a broad term that refers to the allocation of processing resources in space, time and to activities [1]. The allocation of attention in space, referred to as orienting or spatial attention, can be controlled exogenously—or bottom-up—by external stimuli in the environment and endogenously—or top-down—by our internally generated expectations or intentions [2]. Regardless of the mode of control, the distribution of our cognitive resources among the many competing stimuli around and within us controls which stimuli we consciously perceive. When attention is allocated effectively, these should be the stimuli most relevant to us at the moment.

Studies using visual stimuli that are near the threshold of perceptibility (i.e., very brief and/or faint) have found that spatial attention increases the likelihood that a stimulus will be consciously perceived (e.g., Chica et al. [3]), and that this effect is largest when attention is elicited exogenously [4]. This suggests that reflexive spatial attention, and to a lesser extent voluntary spatial attention, improve the likelihood that visual stimuli will be perceived. The effect of attention on the accuracy of perception is less clear: in the same studies as above, spatial attention was found to have little to no effect on the accuracy of responses for stimuli while controlling for self-reported conscious perception. Conversely, studies using stimuli above the threshold of perception have found that spatial attention influences how many properties of stimuli are perceived, including contrast [5,6], colour saturation [7], spatial frequency [8,9], and the size of gaps in line objects [9]. These findings suggest that attention affects how stimuli are perceived, beyond the likelihood that they are perceived at all.

A powerful method for studying the effects of attention generally, and spatial attention specifically, on conscious perception is to use a continuous response variable. Given that many stimulus properties are continuous (e.g., contrast, rotation, tone) and not binary (e.g., present/not present), it is possible to measure responses in a similarly continuous manner. Paradigms using continuous response variables are sometimes referred to as infinite-alternative forced choice (∞-AFC), as participants are able to make responses at any point across a continuous spectrum. This approach has some important advantages over traditional two-alternative forced choice (2-AFC) paradigms, wherein participants are limited to making one of two responses (e.g., “Which stimulus had higher contrast? (Left/Right)”): first, it allows researchers to measure accuracy as a spectrum instead of a hard binary (i.e., correct or incorrect), providing richer data. Second, it allows us to explore how different factors improve or impair the overall accuracy of perception as opposed to how it shifts our thresholds for comparison judgements.

One of the first perception studies to employ an ∞-AFC paradigm was Prinzmetal et al. [10], where the authors used several such measures to investigate the effects of dual-task interference on the accuracy of perception. In one experiment, the authors devised a paradigm where coloured targets (small dots) were presented briefly on either the left or right side of the screen while a second task (a 3 × 3 letter array search) divided their attention, and participants were required to indicate the colour of the target on a colour wheel using the computer cursor. The authors used this paradigm to measure the impact of various manipulations of attention on the accuracy of colour responses, finding that presenting the 3 × 3 search array for the interfering task on the opposite side of the screen as the target, increasing the difficulty of the interfering task, and presenting the interfering task and colour target simultaneously (as opposed to sequentially) all had detrimental effects on the accuracy of responses via the colour wheel. These findings, along with the findings of the other experiments in their study, led Prinzmetal et al. [10] to conclude that accuracy of visual perception was improved by various manipulations of attention.

**Figure 1:**
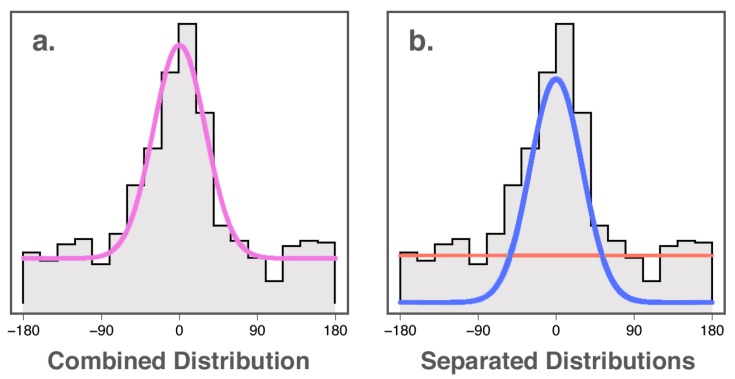
(**a**) combined distribution of random guesses and responses for perceived colours on a colour wheel task; (**b**) separated uniform (red line) and von Mises (blue line) distributions for the same colour wheel response data.

**Figure 2:**
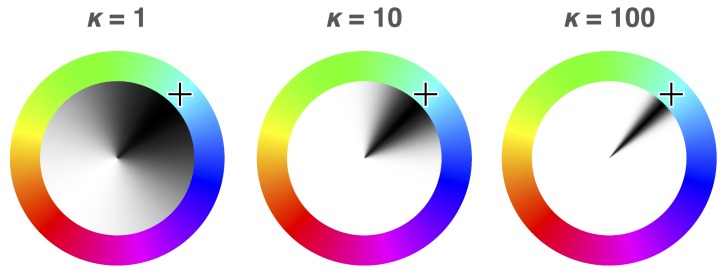
The theoretical distributions of responses on the colour wheel for low, high, and very high fidelities (from left to right), with the densities of the distributions indicated by the shading inside the colour wheels. The concentration of responses around the colour indicated by the cross is indicated in units of kappa (κ) for each wheel, with higher κ values indicating lower variance in responses.

In the years since Prinzmetal et al. [10], new statistical methods have been developed that allow the separation of “accuracy” of colour wheel responses into two sub-components: probability (the likelihood that a stimulus will be perceived), and fidelity (the accuracy with which perceived stimuli are encoded). Interested in the maintenance of colour in working memory, Zhang and Luck [11] developed a mixture-modelling approach to analysing colour wheel data based on the response distributions that would be expected for remembering and blind guessing. If participants retained no memory of the target colour, the authors reasoned that their responses would be expected to be distributed uniformly along the colour spectrum (red line in Figure 1b). If they did remember the target colour, however, responses would be expected to follow a von Mises distribution (normal distribution adjusted for a circle) centred around the location on the colour wheel where the colour of the target was drawn from (blue line in Figure 1b). Thus, the combined distribution of angular error on the colour wheel (Figure 1a) is able to be modelled as being a mixture of these two distributions in some proportion, giving us two parameters: the proportion of the error best explained by blind guessing, and the concentration parameter of the error distribution for non-guessing responses. The probability of memory is defined in this model as the proportion of non-guessing responses. The fidelity of memory is defined in this model as the concentration parameter κ (kappa) of the von Mises distribution of the angular error for non-guessing responses: if responses centred around the true colour have a wide spread, fidelity of memory is inferred to be poor (Figure 2). Likewise, if the distribution of responses is concentrated around the true colour, fidelity of memory is inferred to be high. This method of analysis is elaborated on in Lawrence [12].

Despite being developed to isolate the probability and fidelity of encoding in working memory, the method of analysis developed by Zhang and Luck [11] can likewise be applied to the study of conscious perception. Consider that a colour must first be perceived in order to enter working memory: if factors that affect working memory storage and retrieval (e.g., memory load) are held constant while factors that affect perception (e.g., attention) are manipulated, then any effects of experimental manipulations on the probability or fidelity of responses must therefore be at the perceptual level. Thus, the colour wheel and mixed modelling paradigm can be used to investigate the effects of various types of attention on (a) the likelihood that stimuli will be consciously perceived, and (b) the accuracy with which they are perceived. An important advantage of this method over some used in prior studies is that it does not rely on the accuracy of participants’ meta-awareness of whether they perceived something to determine the probability of perception, instead using statistical inference on the distributions of responses along the colour wheel to determine the proportion of blind guessing.

In recent years, researchers have called the utility of the Zhang and Luck (ZL) model into question in the context of working memory, finding that models accounting for the number of colour stimuli held simultaneously in memory vastly outperform the simple ZL mixture model in terms of model fit [13,14]. Despite this, we believe that the ZL model is well-suited for studying the effects of attention on the perception of colour. In terms of model selection, the working memory models that have been found to outperform the ZL mixture model do so by accounting for stimulus set size in various ways [13], and are thus ill-suited for tasks where only one colour stimulus is presented per trial. Additionally, the ZL model reflects our theoretical expectation that angular error for brief single-colour stimuli will arise from two processes: blind guessing when the colour target is not perceived at all, and imprecision when the colour target is perceived.

To date, little research has been done using the Zhang and Luck [11] colour wheel method to investigate the effects of spatial attention on conscious perception: to our knowledge, the only two attentional studies to use this analysis have been Redden et al. [15] and Redden et al. [16]. These two studies integrated the colour wheel into temporal order judgement (TOJ) tasks as a diagnostic to verify that attention was being biased towards the intended locations in space, and manipulated spatial attention endogenously via between-block instructions that informed participants where colour probes were more likely to appear. Redden et al. [15] found that endogenous attention improved both the probability and fidelity of colour encoding in participants that used the probe bias instructions to direct their attention. Redden et al. [16] likewise found that endogenous attention improved the perception of colour targets, but found that the specific effects on fidelity and probability of encoding varied depending on the colour probe duration. To date, no studies using the mixed-modelling colour wheel method have been done exploring the influence of endogenous or exogenous spatial cues on the perception of colour, nor have any studies explored the effects of exogenous attention on the probability and fidelity of conscious perception. Here, in two experiments, we aim to address these gaps in the literature.

In our first experiment, we used alphanumeric characters as endogenous cues in a Posner-like cueing paradigm [2] to direct attention to the left side, right side, or neither side of the screen, followed by a target at either the left or right location. As such, targets could be presented at the location indicated by the cue (validly cued targets), at the location opposite from that indicated (invalidly cued targets) or following an uninformative cue (neutrally cues targets). Spatial cues were valid on 80% of the trials on which they occurred. The stimulus onset asynchrony between cues and targets (SOA) was 800 ms for all trials in this experiment.

In our second experiment, we used spatially-uninformative bright flashes as exogenous cues in a Posner cueing paradigm otherwise similar to Experiment 1. Past research on exogenous spatial attention has found that spatial cues facilitate responding to targets when cue-target intervals are shorter than 200–300 ms and inhibit responding to targets at longer cue-target intervals. This latter phenomenon is known as “Inhibition of Return” (IOR, see Klein [17] for a review). To explore how IOR affects the perception of colour, we varied the SOA between 100 ms and 800 ms within blocks, giving us a short SOA for which we would expect valid cues to facilitate processing at the cued location, and a long SOA, for which we would expect them to inhibit processing at the cued location. In both experiments, participants were required to make detection responses to targets as quickly as possible, and subsequently indicate the colours of the targets they had detected as accurately as possible on an RGB colour wheel.

Based on the findings of past research, we hypothesized that, for endogenous cues, participants would have faster reaction times to validly cued targets than invalidly cued ones, and that there would be a facilitatory effect at the cued location on at least one of the two properties of colour encoding (probability and fidelity). For exogenous cues, we predicted faster reaction times to targets at the cued location when the SOA was short (facilitation) and slower reaction times when the SOA was long (inhibition). Given the lack of existing literature on the topic, we did not have any concrete hypotheses about how exogenous attention would affect the probability and fidelity of encoding of colour stimuli.

## 2. Experiment 1

### 2.1. Methods

#### 2.1.1. Participants

Participants were recruited from a local undergraduate participant pool and included a total of 81 individuals. All participants gave their informed consent prior to participating, and were compensated via their choice of course credit (one percentage point per hour) or 15$ per hour for their participation. The experiment was conducted in accordance with the Declaration of Helsinki, and ethical approval for the experiment was provided by the Dalhousie University Social Sciences and Humanities Research Ethics Board (file number: R04/05.2011-2496).

Due to variability in the time participants took to complete each trial, 10 participants failed to complete the entire experiment within the allotted time and were consequently removed from analysis. Following exclusions, the data of 71 participants were included in the final analysis (23 male, 7 left-handed, *M_age_* = 20.1, age range: 18–30 years).

#### 2.1.2. Materials

The experiment was coded in the Python programming language using the pyglet library, and run on a Mac Mini computer with a 2 GHz processor running Mac OS X 10.5.7 (Apple Inc., Cupertino, CA). The experiment code, along with animations illustrating the paradigms in action and instructions on how to replicate, can be found on our lab’s GitHub page (https://github.com/TheKleinLab/colour_wheel_cueing). Stimuli were displayed using a 19-inch CRT monitor at a resolution of 1024 × 768 pixels and a refresh rate of 120 Hz. Responses were collected via USB keyboard and mouse.

**Figure 3:**
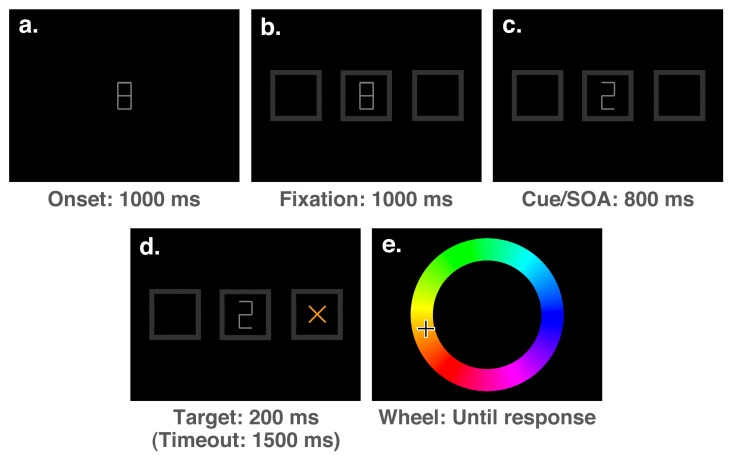
The sequence and duration of events during a single trial for Experiment 1. The sequence of the stimuli is indicated by the letters in the top-left corner. For the purpose of illustration, stimuli are not drawn to scale.

The stimuli presented at each stage of a trial can be seen in Figure 3. Each trial of the experiment started with the presentation of a medium grey (50% white) fixation stimulus (a number “8” drawn with straight lines, measuring 0.375° of visual angle in width and 0.75° in height with a line thickness of 0.05°) at the centre of the screen for a duration of 1000 ms (see Figure 3a). The fixation period was followed by the onset of three dark grey (20% white) boxes aligned horizontally (each measuring 1.5° and with a line thickness of 0.3°): one at the centre surrounding the fixation stimulus, and two peripheral boxes with centres offset from fixation by 7° (Figure 3b). After an interval of 1000 ms, this display was followed by the onset of a cue, which consisted of the removal of either the upper-left and lower-right lines of the “8”, rendering a “2”, the lower-left and upper-right lines, rendering a “5”, or the top and bottom lines, rendering an “H”. The “2” and “5” stimuli were presented as spatial cues on 5 out of every 6 trials, accurately indicating the target location on 4 out of 5 spatial cue trials (i.e., 80% of the time). On the remaining 1/6th of trials, the “H” stimulus was presented as a neutral cue, providing no spatial prediction. Following the cue onset by 800 ms, a target (an “x”, measuring 0.5° with a line thickness of 0.05°) with a colour selected randomly from an RGB colour wheel appeared in either the left or right box and remained on-screen for a duration of 200 ms (Figure 3d). The full colour spectrum from which target colours were selected is illustrated accurately in both Figure 2 and Figure 3e. After a speeded detection response to the target was made by pressing the space bar, or a timeout interval of 1500 ms had elapsed since the target onset, the screen was cleared and an RGB colour wheel (an annulus with an outer diameter of 7.5° and a thickness of 2.2°, with angle of rotation varying randomly between trials) and mouse cursor were presented on the screen (Figure 3e). The screen was cleared once a location on the colour wheel had been clicked, ending the trial.

On 20% of trials no target was presented, serving as catch trials to discourage anticipatory responding. Thus, 6 (4 valid cues + 1 invalid cue + 1 neutral cue) × 2 (target location) × 5 (20% catch trials) = 60 trials were necessary to complete the design.

#### 2.1.3. Procedure

After participants had read and signed the consent forms for the study, participants were given verbal instructions on how to perform the task. The mapping of numeric cues to locations in space was counterbalanced between participants: half the participants were instructed that the central cue “2” meant that the target would likely appear on the right and “5” indicated it would likely appear on the left, and the other half were told the inverse. All participants were instructed that, if an “H” appeared, the target was equally likely to appear on the left or right.

Participants completed seven test blocks of 60 trials each, for a total of 420 trials. Prior to the test blocks, participants completed a practice block consisting of 30 trials randomly selected from the full 60 of a test block. Participants were allowed to take a break every 30 trials.

### 2.2. Results

All analysis was performed using version 3.5.1 of the R statistical software [18], in conjunction with the Stan platform for Bayesian statistical modelling [19] via RStan 2.18.2. In the interest of reproducible science, the raw data and analysis scripts necessary to reproduce the results and visualisations reported in this manuscript have been made openly available via the Open Science Framework, as described in the Supplementary Materials.

A hierarchical Bayesian mixed-effects model was used for data analysis, the details of which can be found in Appendix A. Bayesian statistics is an approach that aims to approximate the probability distributions of one’s parameters of interest. This is in contrast to frequentist statistics, where statistical inference is typically made based on the probability of obtaining one’s current data with repeated observations, assuming that the null hypothesis is true (see Chapter 1 of McElreath [20] for a more comprehensive explanation). We selected a Bayesian model for our data because we believe it to be a more robust and suitable method of implementing the Zhang and Luck [11] mixture model analysis because it avoids many of the problems inherent in frequentist statistics, and because we believe the results of a Bayesian model to be more intuitive to interpret than those of traditional statistical tests. We used a hierarchical model because it accounts for between-subject variation and makes the results robust against differing trial counts between participants.

Because the Bayesian approach gives us distributions of probability for our estimates of parameters and effects (known as Bayesian posterior distributions), the way in which we interpret them differs from how we interpret frequentist results. For example, we are not beholden to the black-and-white decision-making of the Neyman–Pearson framework, which requires that we choose the rate at which we want to obtain false positives across our careers (typically 0.05, or 95% confidence), then reject the null hypothesis whenever our *p*-value is less then our cutoff and accept the null when the *p*-value is greater than it. Instead, because posterior distributions represent the probability of the parameter of interest being within a given range of values, we can instead talk about the credibility of effects.

In frequentist statistics, confidence intervals counter-intuitively represent the interval that, if calculated for repeated hypothetical samples, would contain the true parameter value a certain percentage of the time (e.g., 95% of the time for a 95% CI). In this study, we will instead discuss our findings in terms of Highest Density Intervals (HDIs), which represent the range of values that we believe contain the true population mean with a certain level of likelihood, given our data. Because a difference between distributions of zero represents the absence of a cueing effect, we will refer to effects as “highly credible” when their 95% HDI does not contain zero, “weakly credible” when their 95% HDI contains zero but their 50% HDI does not, and non-credible when their 50% HDI contains zero.

The probability distributions for the effects of cueing (valid versus invalid) and values of the parameters across cueing conditions are visualised using “violin plots”, which represent the shape of the probability distribution for a given parameter or effect along the *x*-axis. That is, the width of the shape at any given value on the *y*-axis represents how likely that value is to be the true population mean. In addition, each “violin” contains a black dot representing the median value, a thin black line representing the range covered by the 95% HDI, and a thicker black line representing the range covered by the 50% HDI.

After excluding participants who had not completed all blocks of the experiment, data was pre-processed prior to modelling with Stan. All practice blocks were dropped from the data, as were all trials on which participants failed to make a detection response to targets (misses, 0.32% of target trials), made detection responses on catch trials where no targets were presented (false alarms, 3.02% of catch trials), or made detection responses on target trials before targets appeared (pre-target responses, 1.22% of target trials). Reaction times, which were right-skewed, were log-transformed to make their distribution approximately normal. For each participant, the median absolute deviation around the median (MADM) for log reaction times for each cue condition was computed, and any trials in that condition with log(rt)s that deviated from the median by more than five times the MADM were considered outliers and excluded from subsequent analysis. Using this procedure, 2.37% of the remaining trials were excluded.

**Figure 4:**
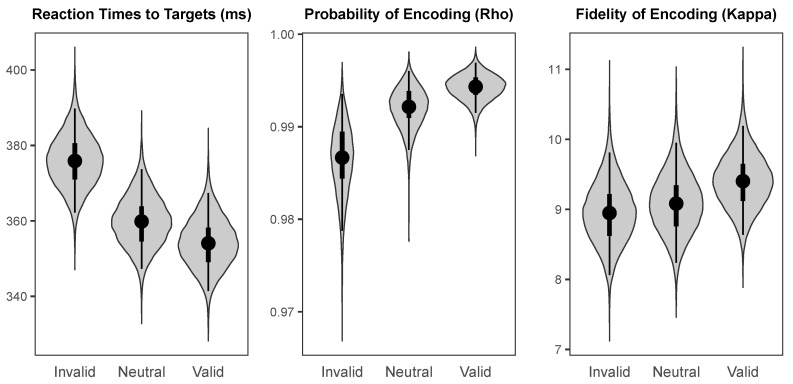
The probability distributions, medians, and HDIs for the population means of the parameters of interest for each cue condition for endogenous cues.

The probability distributions for the parameters of interest in each cueing condition are shown in Figure 4. The median intercept for reaction times to targets was 359.86 ms (*HDI_95%_* = [347.30 ms, 373.70 ms]). The intercept for probability of colour encoding was near-ceiling (Median = 0.992, *HDI_95%_* = [0.987, 0.996]), and the intercept for fidelity of colour encoding was likewise high (Median = 9.08, *HDI_95%_* = [8.24, 9.95]).

**Figure 5:**
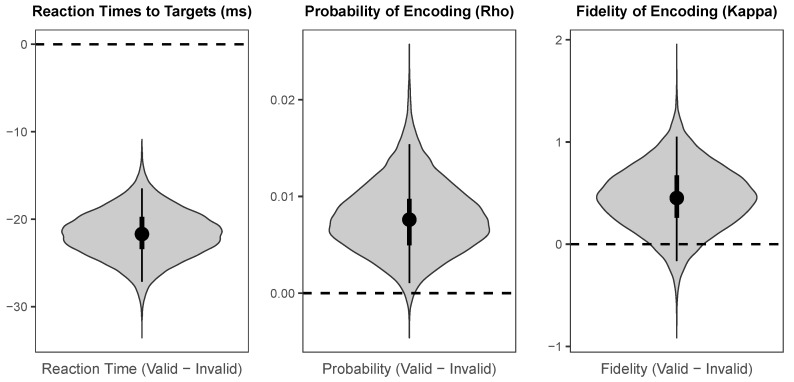
The probability distributions for the effects of cueing (Valid minus Invalid) for each parameter of interest for endogenous cues.

As illustrated in Figure 5, there was a large credible benefit of cueing on reaction times to targets (Median = −21.70 ms, *HDI_95%_* = [−27.15 ms, −16.47 ms]), a small credible benefit of cueing on probability of colour encoding (Median = 0.008, *HDI_95%_* = [0.001, 0.015]), and a small weakly credible benefit of cue validity of fidelity of colour encoding (Median = 0.45, *HDI_95%_* = [−0.17, 1.05], *HDI_50%_* = [0.26, 0.67]).

### 2.3. Discussion

In agreement with a substantial literature, the results from the speeded detection responses demonstrate that endogenous spatial attention speeds target detection. Moreover, the mixed-model analysis of the colour wheel response data found that valid endogenous cues credibly improved the probability that target colours were perceived at all, despite the overall probability of colour perception being near–ceiling. Additionally, our model provides some limited evidence that valid endogenous cues improved the fidelity with which colours were encoded. This pattern of results is consistent with the findings of Redden et al. [15] and Redden et al. [16], both of which found that biasing attention to one of two target locations in TOJ tasks using between-block instructions improved the probability and/or fidelity of the encoding of colour information. Building on these papers, our study provides the first evidence for the effects of endogenous spatial cueing on the probability and fidelity of colour encoding. These results are consistent with the finding that spatial attention alters the sensitivity of neurons in the V4 region of the visual system [21,22], which is the region largely responsible for the perception of colour [23].

## 3. Experiment 2

### 3.1. Methods

#### 3.1.1. Participants

Forty-two participants were recruited from a local undergraduate participant pool. All participants gave their informed consent prior to participating, and were compensated via their choice of course credit (one percentage point per hour) or 15$ per hour for their participation. The experiment was conducted in accordance with the Declaration of Helsinki, and ethical approval for the experiment was provided by the Dalhousie University Social Sciences and Humanities Research Ethics Board (file number: R04/05.2011-2496). Due to variability in the time participants took to complete each trial, 2 participants failed to complete the entire experiment within the allotted time and were consequently removed from analysis. One participant completed an additional 54 non-practice trials beyond the full count due to a relaunch of the experiment program partway through the session, all data from which was included in the final analysis (because hierarchical models are robust against participants with varying trial counts, inclusion of this data did not violate any of our statistical assumptions). Following exclusions, the data of 40 participants were included in the final analysis (8 male, 5 left-handed, *M_age_* = 19.5, age range: 18–25 years).

#### 3.1.2. Materials

**Figure 6:**
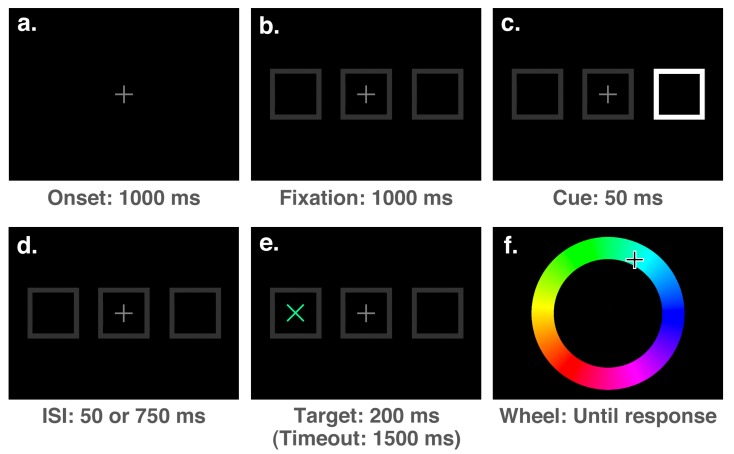
The sequence of events on a single trial for Experiment 2. The sequence is indicated by the letters in the top-left corners of the boxes, and show the stimuli presented during an invalidly cued trial. For the purpose of illustration, stimuli are not drawn to scale.

The stimuli presented at each stage of a trial are shown in Figure 6. Materials were the same as in Experiment 1 with the exception of the fixation and cue stimuli. In Experiment 2, the fixation stimulus was a cross measuring 0.5°, with a line thickness of 0.05° (see Figure 6a). Cue stimuli consisted of a 50 ms change of one of the boxes to 100% white, as shown in Figure 6c.

The onsets of the cue and target were separated by a stimulus-onset asynchrony (SOA) of either 100 ms or 800 ms (depending on the trial), equivalent to inter-stimulus intervals (ISIs) of 50 ms or 750 ms, respectively (see Figure 6d). Spatial cues were entirely uninformative, with an equal number of valid, invalid, and neutral cues per block. Apart from these differences, the sequence of events for trials in Experiment 2 were the same as in Experiment 1. On 20% of trials no target was presented, serving as catch trials to discourage anticipatory responding. Thus, each block consisted of 60 trials (3 cue locations × 2 target locations × 2 SOA lengths × [4 target trials + 1 catch trial]).

#### 3.1.3. Procedure

Once participants had read and signed their consent forms, they received verbal instructions on how to perform the task. Participants were instructed to respond to targets as quickly as possible, and to try and maintain their gaze on the fixation stimulus while it was present, using their peripheral vision to detect cues and targets. Participants were seated 60 cm from the screen and instructed to maintain this distance by checking their position using a measured length of string during each break in the experiment.

Each session consisted of eight test blocks of 60 trials each, for a total of 480 trials. The order of trials was randomized within each block, and participants were provided the opportunity to take a break every 30 trials. Prior to beginning the eight blocks of test trials, participants also completed 30 practice trials that were sampled randomly from the 60 trials of the complete design. Each session of the experiment took approximately 60 min to for participants to complete.

### 3.2. Results

The data from Experiment 2 was analysed similarly to Experiment 1 with a Bayesian mixed-model (see Appendix B for more detail). Preprocessing of data prior to modeling with Stan was likewise similar: all practice blocks were dropped from the data, as were all trials on which participants failed to make a detection response to targets (misses, 0.74% of target trials), made detection responses on catch trials where no targets were presented (false alarms, 2.67% of catch trials), or made detection responses on target trials before targets appeared (pre-target responses, 1.47% of target trials). Additionally, for each participant, the median absolute deviation around the median (MADM) for log reaction times for each (SOA × cue condition) combination was computed, and any trials in that condition combination with log(rt)s that deviated from the median by more than five times the MADM were considered outliers and excluded from subsequent analysis. Using this procedure, 2.28% of the remaining trials were excluded.

**Figure 7:**
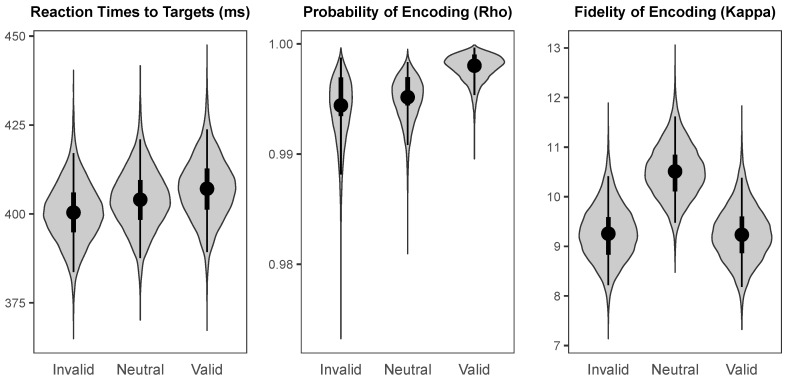
The probability distributions for the population means of the parameters of interest for each cueing condition at the short (100 ms) SOA.

**Figure 8:**
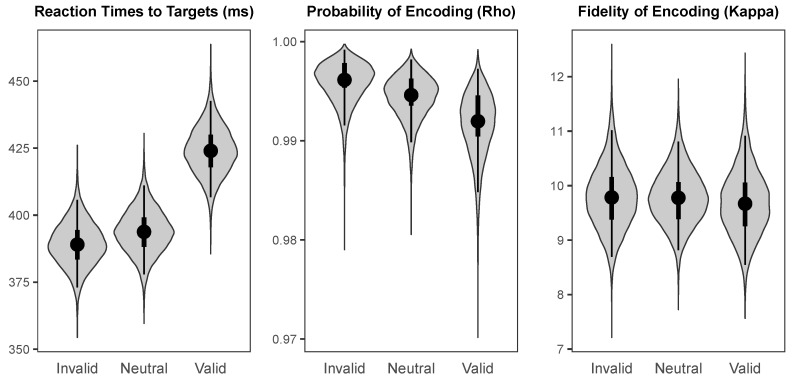
The probability distributions for the population means of the parameters of interest for each cueing condition at the long (800 ms) SOA.

The probability distributions for the parameters of interest across cueing conditions at the 100 ms and 800 ms SOAs are illustrated in Figure 7 and Figure 8, respectively. Reaction times to targets had a median intercept of 404.02 ms (*HDI_95%_* = [387.63 ms, 420.96 ms]) at the 100 ms SOA and 393.78 ms (*HDI_95%_* = [377.95 ms, 411.07 ms]) at the 800 ms SOA. Probability of colour encoding was near-ceiling at both the 100 ms SOA (Median = 0.995, *HDI_95%_* = [0.991, 0.998]) and the 800 ms SOA (Median = 0.995, *HDI_95%_* = [0.990, 0.998]). Fidelity of colour encoding was likewise high at both the 100 ms SOA (Median = 10.51, *HDI_95%_* = [9.48, 11.62]) and the 800 ms SOA (Median = 9.78, *HDI_95%_* = [8.81, 10.81]).

**Figure 9:**
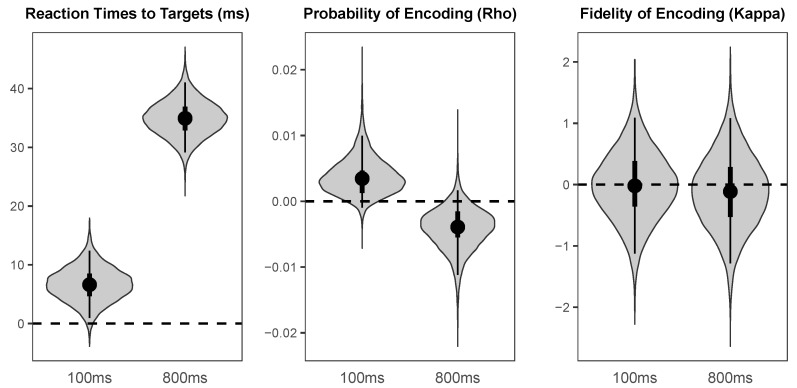
The probability distributions for the effects of cue validity (Valid minus Invalid) for each parameter of interest at the short (100 ms) and long (800 ms) SOAs.

The effects of valid versus invalid cueing on the parameters of interest at the short and long SOAs are illustrated in Figure 9. At the 100 ms SOA, there was a small but highly credible cost of cue validity on reaction times to targets (Median = 6.62 ms, *HDI_95%_* = [0.95 ms, 12.41 ms]), a small weakly credible benefit of cueing on probability of colour encoding (Median = 0.003, *HDI_95%_* = [−0.001, 0.010], *HDI_50%_* = [0.001, 0.005]), and no credible effect of cueing on fidelity of colour encoding (Median = −0.02, *HDI_50%_* = [−0.36, 0.39]). At the 800 ms SOA, there was a large highly credible cost of cueing on reaction times to targets (Median = 34.94 ms, *HDI_95%_* = [29.14 ms, 41.05 ms]), a small weakly credible cost of cue validity on probability of colour encoding (Median = −0.004, *HDI_95%_* = [−0.011, 0.002], *HDI_50%_* = [−0.005, −0.002]), and no credible effect of cue validity on fidelity of colour encoding (Median = −0.11, *HDI_50%_* = [−0.53, 0.29]). Although following a spatial cue there was no credible benefit for targets presented at the cued relative to the uncued location cues on fidelity of encoding at either SOA, there was a highly credible benefit following a neutral cue on fidelity of encoding relative to either valid or invalid peripheral cues at the 100 ms SOA (Median = 1.26, *HDI_95%_* = [0.44, 2.12]).

### 3.3. Discussion

At the long SOA, we observed the predicted IOR effect, with reaction times being considerably slower for targets presented at cued relative to uncued or neutrally cued targets. Consistent with this pattern, we also found a weakly credible cost at the cued location on the probability of colour encoding, suggesting that the mechanisms responsible for IOR similarly reduce the probability of perceiving colour stimuli. There were no credible effects of cueing on the fidelity of perception at the long SOA. This pattern of results is consistent with our hypothesis, which was that exogenous cueing would have an effect on at least one of the colour perception measures, and that it would be consistent with the pattern of reaction time results.

At the short SOA, however, we observed a small-but-credible cost of valid cueing on reaction times at the short SOA. In addition, this effect did not match the pattern of cueing effects for the colour perception data at the short SOA, where cueing had a weakly credible benefit for probability of colour encoding and no effect on its fidelity. Moreover, we found a highly credible reduction in the fidelity of colour encoding for targets preceded by spatial cues relative to neutral cues at the short SOA, which differs from the patterns with reaction time and probability at this SOA.

The pattern of results observed at the short SOA is surprising for several reasons: first, we observed a cost of cueing on reaction times instead of the facilitation we expected. Second, this cost did not match with a corresponding effect on either measure of colour perception. Third, the two measures of colour perception were not affected by cueing in the same manner, with probability and fidelity having very different patterns of effects across the three cue conditions.

Although facilitation of reaction times is generally observed at short SOAs in exogenous cueing paradigms, this finding is not universal. As summarized in Collie et al. [24], there have been several spatial cueing studies in which valid exogenous cues have failed to produce facilitation at SOAs under 200 ms (e.g., Tassinari et al. [25]). Moreover, Milliken et al. [26] found that valid exogenous cues only speeded reaction times relative to invalid cues for 2-AFC discrimination responses, finding no early facilitation for detection responses. We believe that the slower reaction times we observed to validly cued targets at the short SOA might have been due to an onset-detection cost for validly cued targets [27]. In other words, the onset of the bright exogenous cues just prior to the presentation of the smaller, thinner colour targets may have slowed detection of targets (and thus reaction times) when they appeared at the cued location. This account might explain why valid cueing improved the probability of encoding the colours of targets despite slowing their detection, since it holds that, when valid, cues would have oriented attention to the locations of targets despite the reaction time cost.

The reasons for the unexpected costs of spatial cueing compared to neutral cueing on the fidelity of colour encoding at the short SOA are more difficult to explain: it may be the case that our neutral cues were not truly neutral, and improved the fidelity of colour perception in a way that the spatial cues did not. Alternatively, it might be that both types of spatial cueing impaired the fidelity of colour perception relative to neutral cues. Future research should attempt to replicate and explain this finding in greater detail. In summary, whereas we did find evidence of inhibition of return at the long SOA, we did not find evidence of early facilitation for either detection RT or fidelity of perception.

## 4. General Discussion

Together, our findings from Experiments 1 and 2 provide preliminary evidence that both endogenous and exogenous cueing influence the accuracy with which colour information is encoded into conscious awareness. Specifically, we found that endogenous spatial cueing improved both the probability and fidelity of colour encoding, whereas exogenous spatial cueing appears to affect probability but not fidelity. Although these effects were small, and additional research is needed to increase our confidence in these findings, our Bayesian models determined our observed effects to be weakly-to-highly credible and thus deserving of consideration. Additionally, our experiments showcase the utility of using continuous response variables in attention research, which can provide rich information for answering questions about how various factors influence the encoding of stimuli.

In the introduction, we noted that if factors affecting working memory storage and retrieval are held constant, then we can attribute the effects of our attentional manipulation to perception. In both of our experiments, however, the interval between target presentation and colour wheel onset varied depending on how quickly participants made a detection response to each target. As pointed out by an anonymous reviewer, this raises the possibility that our observed effects on probability and/or fidelity could be due to memory rather than perception, because slower detection responses in some cue conditions would result in greater opportunity for decay of colour information already encoded into working memory. For a variety of reasons, we believe that this is highly unlikely. Firstly, with a single colour stimulus per trial to hold in memory and brief intervals between targets and responses, decay seems unlikely. Secondly, the appearance of the colour wheel ~400 ms (the speed of the typical detection response) after the target provided the opportunity to make the colour response, but this response was usually made a second or more later. Hence, relative to the overall time between target removal and colour response, the RT differences (10 ms to 35 ms) provide little opportunity for differences in decay. Thirdly, the pattern of results at the 100 ms SOA in E2 demonstrated a dissociation between reaction times, probability, and fidelity, which would not be expected if our probability and fidelity effects were due to differences in colour decay associated with mean reaction times. Finally, the previously mentioned TOJ studies [15,16] found effects of attention on the probability and fidelity of colour perception in paradigms that held the target-response interval constant across trials, providing precedent for effects of attention on colour perception unrelated to working memory.

In discussing the study of the effects of attention on conscious experience, Shulman [28] recommended that researchers use paradigms that allow the isolation of the effects of attention on different perceptual processes in order to better understand the interactions between attention and perception. By allowing researchers to separately measure the effects of attention on the probability and fidelity of colour encoding, the mixed-modeling colour wheel paradigm does exactly this, and provides attention researchers with a powerful, information-rich tool for studying visual perception.

Across both experiments, we found the overall probability of perception to be near-ceiling and the fidelity of perception to be fairly high. Although it is interesting to study the effects of attention on perception in all contexts, a paradigm in which overall perceptual performance was lower might show larger or clearer effects of attentional manipulations on the probability and fidelity of perception. There are a number of possible ways this might be done, including making targets smaller, more brief, or presented at a greater eccentricity from fixation. Additionally, if the effects of an attentional manipulation happen at the locus of decay in working memory (i.e., attention reduces the decay of perceived stimuli), increasing the target-wheel interval to at least 1000 ms might help ensure sufficient decay to detect such effects.

## 5. Conclusions

The experimental and analytic methods used here are applicable far beyond the context of a Posner cueing paradigm. The colour wheel paradigm (or any similar one using a continuous response), for example, could be used in conjunction with any attentional manipulation (e.g., alerting, dual-task interference) that uses a briefly-presented visual target. Similar response measures and analyses for studying the effects of attentional manipulation on the probability and fidelity of brightness, contrast, gap size, or saturation perception would also be simple to implement, and offer novel insight into how different forms of attention affect our conscious experience of the world around us.

## Supplementary Materials

The data and analysis scripts from both experiments, along with the files necessary to compile the manuscript, are available online via the Open Science Framework: https://osf.io/kmzxn/.

## Abbreviations

The following abbreviations are used in this manuscript:

IOR	Inhibition of Return
SOA	Stimulus Onset Asynchrony
HDI	Highest Density Interval
TOJ	Temporal Order Judgement

## Appendix A

The Bayesian model for Experiment 1 was written in the Stan language. Log-transformed reaction times were modeled as being sampled from a normal distribution. Angular errors were modeled as being sampled from a mixture model combining a uniform distribution representing random guessing and a von Mises distribution centered on 0 representing the distribution of non-random responses, yielding two parameters: probability of encoding (ρ, the proportion of the von Mises component of the mixture), and fidelity of encoding (κ, the spread of the von Mises distribution). Thus, reaction times and angular error were modeled as:

$$\begin{array}{c} \log(rt)∼\mathrm{Normal}(\mu ,\sigma )\\ \mathrm{AngleErr}∼(1-\rho )\cdot \mathrm{Uniform}+(\rho )\cdot \mathrm{VonMises}(0,\kappa ) \end{array}$$

Estimates of ρ and κ within the model were transformed to logit(ρ) and log(κ), respectively, such that their distributions were approximately normal. The model was hierarchical such that the intercepts and cueing effects for all parameters were estimated for each participant, allowing the model to account for between-subject variability in responses. A correlation matrix was included in the model to allow any associations between parameters or effects to be measured. Weak uninformative Student T priors were used for all parameter intercepts and cueing effects.

Samples from the posterior distribution for Experiment 1 were drawn using the default No U-Turn Sampler (NUTS) in RStan 2.18.2. The sampler ran 10 chains collecting 6000 samples from the posterior each, half of which were discarded as warm-up samples, resulting in 30,000 total samples for our analysis. Diagnostics on the model revealed that convergence between chains was adequately high (all R-hat ≈ 1.0), and that the effective sample size for all parameters (N_effective_) was greater than 1000 for all estimated parameters. The obtained posterior probability distributions for reaction times, ρ, and κ were all back-transformed from their normalized forms to their original units prior to the generation of HDIs and plots.

## Appendix B

The Bayesian model for Experiment 2 was the same as the model for Experiment 1 apart from the addition of the SOA factor, such that the effects of cue validity, SOA length, and the interactions between the two were modeled for each parameter. Additionally, due to the increased complexity of the model relative to the model for Experiment 1, the correlation matrix had to be removed in order for the sampler to be able to achieve numerical stability, and the maximum tree-depth of the model was increased from the default of 10 to 15.

Samples from the posterior distribution for Experiment 2 were drawn using the default No U-Turn Sampler (NUTS) in RStan 2.18.2. The sampler ran 10 chains collecting 4000 samples from the posterior each, half of which were discarded as warm-up samples, resulting in 20,000 total samples for our analysis. Diagnostics on the model revealed that convergence between chains was adequately high (all R-hat ≈ 1.0), and that the effective sample size for all parameters (N_effective_) was greater than 1000 for all estimated parameters. The obtained posterior probability distributions for reaction times, ρ, and κ were all back-transformed from their normalized forms to their original units prior to the generation of HDIs and plots.
